# Extensively drug-resistant and heat-resistant *Enterococcus faecalis* and *Enterococcus faecium* in ready-to-eat meat products

**DOI:** 10.1038/s41538-026-00790-y

**Published:** 2026-03-27

**Authors:** Sara Abdelnaby Sallam, Samir Mohammed Abd-Elghany, Khalid Ibrahim Sallam

**Affiliations:** Department of Food Hygiene, Safety, and Technology, Faculty of Veterinary Medicine, Mansoura University, Mansoura, Egypt

**Keywords:** Microbiology, Molecular biology

## Abstract

The presence of extensively drug-resistant and heat-resistant *Enterococcus faecalis* and *Enterococcus faecium* in ready-to-eat meat products poses a significant public health risk due to their ability to survive thermal processing, persist in the food chain, and disseminate antimicrobial resistance, thereby increasing the risk of foodborne transmission and limiting treatment options. This study evaluated the prevalence, heat-resistance, virulence, and antimicrobial resistance profile of *Enterococcus* isolated from ready-to-eat (RTE) meat products in Mansoura, Egypt. All (100%, 135/135) examined RTE samples (45 each of shawarma sandwiches, Hawawshi, and pastrami slices) were contaminated with *Enterococcus*. PCR targeting the *sodA* gene verified that 63.3% (171/270) of *Enterococcus* isolates were *E*. *faecium* and 36.7% (99/270) were *E*. *faecalis*. The *gelE* and *ace* virulent genes were detected in 71.1% and 65.2% of *Enterococcus* isolates, respectively. Absolute resistance (100%) of enterococcal isolates was found towards penicillin and imipenem, while 86.7%, 85.6%, and 41.9% were resistant to rifampin, vancomycin, and amoxicillin, respectively. All isolates were resistant to at least 4 antibiotics, and 96.3% were resistant to at least 6 antibiotics, with an average MAR index of 0.604. Interestingly, *Enterococcus* in RTE meat samples passed thermal microwave deactivation for 5 minutes, indicating a potent heat stability of this microorganism. The threat caused by virulent, heat-tolerant, vancomycin-resistant enterococci contaminating meat products highlights the risk associated with these pathogens. This emphasizes the urgent need for preventive measures and global strategies to control these emerging foodborne threats.

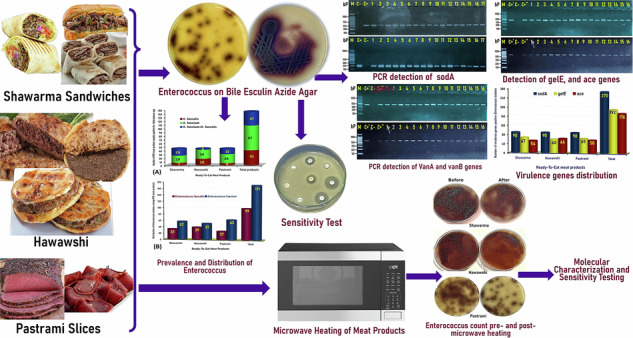

## Introduction

Meat is an excellent source of highly valuable and essential nutrients for the body’s growth and overall health, as it is rich in high-quality protein, vitamins, especially B12, and key minerals, including iron and zinc^1^. This nutritional value, combined with the demands of a fast-moving world marked by rapid tourism growth and lifestyles that keep people away from home, has contributed to the rise of fast-food centers, where ready-to-eat (RTE) meat products are widely sold as convenient “take-away or travel food.” These products are widely consumed without further cooking and are therefore particularly vulnerable to microbiological contamination if hygienic practices during processing, handling, or storage are inadequate.

Among RTE meat products widely consumed in Egypt are Shawarma, Hawashi, and pastrami. Shawarma is a Middle Eastern dish made of thinly sliced meat stacked on a vertical rotisserie and served in pita or flatbread with toppings, while Hawawshi is an Egyptian food of spiced minced meat stuffed in bread and baked until crispy. Pastrami is a seasoned, smoked, steamed deli meat, usually made from beef. The meat of these RTE products in Egypt is mainly derived from cattle or buffalo carcasses. Due to the unhygienic conditions behind RTE meat products, extensive manual handling, prolonged exposure to the environment, and variable temperature control during preparation and retail display, RTE meat products may become contaminated with a wide range of microorganisms, posing potential food safety and public health concerns^2^.

Among these microorganisms, *Enterococcus* species have gained increasing attention due to their dual role as commensal bacteria and opportunistic pathogens. *Enterococcus* spp. are Gram-positive, facultative anaerobic bacteria that naturally inhabit the gastrointestinal tract of humans and animals and are also widely distributed in soil, water, and food environments^3^. In healthy individuals, enterococci are generally harmless members of the intestinal microbiota; however, under certain conditions, particularly in hospitalized or immunocompromised individuals, they can cause severe opportunistic infections^3^. Clinically, *Enterococcus faecalis* and *Enterococcus faecium* are the two species most frequently associated with human disease, including urinary tract infections, bacteremia, endocarditis, wound infections, intra-abdominal infections, and other healthcare-associated infections^4–6^.

Unlike classical foodborne pathogens such as *Salmonella spp*. or *Staphylococcus aureus*, *Enterococcus spp*. are not typically associated with acute food poisoning caused by toxin production. Instead, their relevance in food safety lies in their role as indicators of fecal contamination, their ability to survive adverse environmental conditions, and their capacity to act as reservoirs and vectors for antimicrobial resistance and virulence determinants. RTE meat products are especially susceptible to contamination by enterococci during slaughtering, processing, slicing, storage, and retail handling. Fecal contamination during carcass dressing, inadequate sanitation of equipment and surfaces, and poor personal hygiene of food handlers are major sources of contamination. Furthermore, Enterococcus spp. exhibit exceptional tolerance to environmental stresses, including high salt concentrations, refrigeration, vacuum packaging, and heat exposure, enabling their survival and persistence in processed meat products.

The pathogenic potential of enterococci is further enhanced by the presence of virulence factors that facilitate colonization, persistence, and immune evasion^7^. These factors include surface adhesins, extracellular enzymes, and biofilm-associated proteins, many of which are encoded by genes located on plasmids or pathogenicity islands^7,8^. Key virulence determinants in *E*. *faecalis* and *E*. *faecium*, including the enterococcal surface protein (Esp), gelatinase (GelE), collagen-binding protein (Ace), hyaluronidase (Hyl), and manganese-dependent superoxide dismutase (SodA), have been implicated in biofilm formation, tissue invasion, oxidative stress resistance, and host colonization^9,10^. Additionally, extracellular active gelatinase (*gelE*) is an essential virulence factor linked to *Enterococcus* virulence since it aids in immune evasion by degrading host proteins and antimicrobial peptides^11^, and its presence is linked to increased biofilm formation^12^. Moreover, the collagen-binding protein (Ace) is a cell surface protein that facilitates bacterial attachment to host cell matrix protein and is linked to *Enterococcus* virulence^13^. *Enterococcus* poses serious risks, especially to immunocompromised patients, causing severe infections^14^. The coexistence of virulence traits with multidrug resistance further amplifies the public health significance of enterococci detected in food.

The clinical significance of enterococci is strongly linked to their remarkable intrinsic and acquired resistance to antimicrobial agents. Enterococci exhibit intrinsic resistance to several antibiotic classes, including cephalosporins and low levels of aminoglycosides, largely due to low-affinity penicillin-binding proteins and limited drug uptake mechanisms. Moreover, enterococci have a pronounced ability to acquire and disseminate resistance genes via horizontal antibiotic resistance gene transfer, contributing to the global emergence of multidrug-resistant (MDR) strains not only threaten public health but can have a high capability to transfer genes, which can occur in humans, animals, the environment, or food, particularly ready-to-eat meats and dairy, which can act as reservoirs for antibiotic-resistant strains, and this makes infections harder to treat and control^15–18^. These enterococcal strains often show resistance to glycopeptides such as vancomycin and teicoplanin, as well as aminoglycosides^19^. The first vancomycin-resistant *Enterococcus* (VRE) strains were not identified until 15 years after vancomycin’s introduction in clinical practice in 1972, with a notable increase in VRE cases reported between 1989 and 1993^20^. Of particular concern, infections with glycopeptide-resistant enterococci (GRE), such as VRE, significantly prolong in-hospital stay, increase treatment costs, raise treatment failure risk by 20%, and increase mortality from 27% to 52%, highlighting the serious threat posed by antibiotic resistance ^21–23^

Multidrug-resistant *Enterococcus* strains are capable of causing severe infections, making them a serious threat to public health. The detection of multidrug-resistant and heat-resistant Enterococcus spp. in RTE meat products, therefore, represents a potential public health risk, not due to classical foodborne intoxication, but through their capacity to colonize the human gastrointestinal tract and contribute to the wider dissemination of antimicrobial resistance and virulence genes. Continuous surveillance of these organisms in foods of animal origin is essential for understanding their epidemiology and assessing their impact on food safety and public health.

Accordingly, the present study aimed to investigate the occurrence of *Enterococcus faecalis* and *Enterococcus faecium* in selected ready-to-eat meat products, including shawarma sandwiches, Hawawshi, and pastrami, marketed in Mansoura City, Egypt. In addition, the study evaluated their antimicrobial resistance profiles and thermal tolerance characteristics in order to assess the potential public health risks associated with the consumption of these widely consumed foods.

## Results and Discussion

### Prevalence of *Enterococcus* species in shawarma sandwiches, Hawawshi, and pastrami

**Fig. 1:**
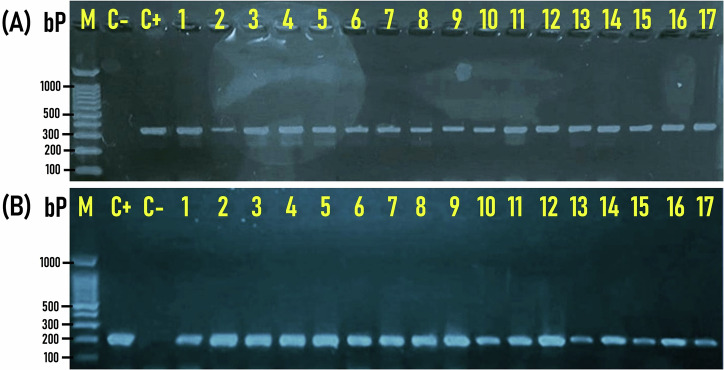
Genetic identification and differentiation of *Enterococcus* isolates. Representative agarose gel electrophoresis for PCR demonstrated the amplified bands of the *SodA* (*Enterococcus faecalis*) and *SodA* (*Enterococcus faecium*) genes (Lanes 1-17), detected at the expected molecular size of 360 bp specific for *Enterococcus faecalis* (**A**) and 215 bp specific for *Enterococcus faecium* isolates recovered from RTE meat products. M: DNA marker (100 bp gene ladder). C+: Control positive (*E*. *faecalis* ATCC 29212 (**A**) and *E*. *faecium* ATCC 19434 (**B**) strains), C–: Control negative (*Escherichia coli K*-12 DH5α). Eight microliters of the PCR product were separated by electrophoresis on a 1.5% agarose gel and visualized under UV light.

In the current study, *Enterococcus* species were detected in 100% (135/135) of ready-to-eat meat products examined, including shawarma sandwiches, Hawawshi, and pastrami slices, with a prevalence rate of 100% (45/45) of each. The 270 isolates of biochemically identified *Enterococcus* spp. (90 from each of the shawarma sandwiches, Hawawshi, and pastrami slices) were further verified and differentiated into *Enterococcus faecalis* and *Enterococcus faecium* by PCR for detection of superoxide dismutase; *sodA* (*E. faecalis*) and *sodA* (*E. faecium*) marker genes, which were detected at the expected molecular size of 360 bp, verifying the *E. faecalis* (Fig. 1A) and 215 bp, verifying the *E. faecium* (Fig. 1B). Of the 270 isolates tested, 99 (36.7%) were molecularly verified as *E. faecalis*, and 171 (63.3%) were verified as *E. faecium*.

The high contamination levels of RTE meat products with *Enterococcus* spp. in the present study are a significant concern, as these products do not undergo further heat treatment after their purchase as sandwiches or slices by the consumers. Enterococci are among the most thermotolerant non-sporulating bacteria, with some strains capable of surviving pasteurization temperatures. Their tolerance to environmental extremes enables them to persist through cooked meat processing and even to multiply during fermentation. Moreover, poor hygienic processing, contaminated surfaces and equipment, unhygienic food handlers, inadequate sanitation of packaging materials, contaminated additives and spices, post-processing contamination, and poor storage conditions are considered major factors contributing to the contamination of the RTE meat products with *Enterococcus* spp., especially *E*. *faecalis* and *E*. *faecium*^24^.

**Fig. 2:**
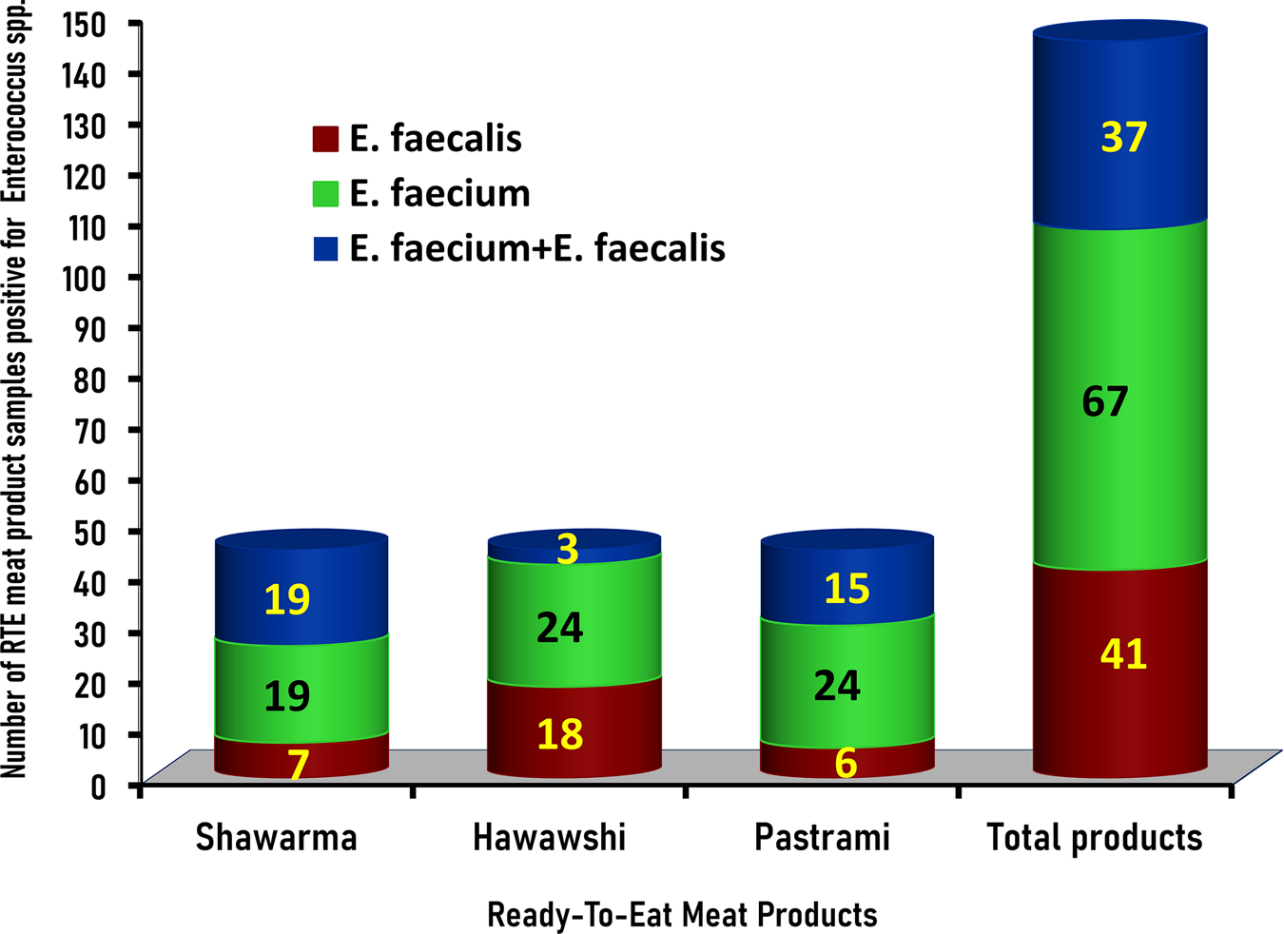
Prevalence of *Enterococcus faecalis* and *Enterococcus faecium* among RTE meat product samples, showing the number of samples positive for *E. faecalis and E. faecium* individually or collectively.

The molecular verification and differentiation of *Enterococcus* isolates indicated that 41 (30.4%), 67 (49.6%), and 37 (27.4%) of the 135 RTE meat products tested were positive for *E. faecalis*, *E. faecium*, and mixed contamination with both *E. faecalis* & *E. faecium*, respectively (Fig. 2). Precisely, 7 (15.6%), 19 (42.2%), and 19 (42.2%) of the 45 tested Shawarma sandwiches; 18 (40%), 24 (53.3%), and (3 6.7%) of the 45 tested Hawawshi sandwiches; and 6 (13.3%), 24 (53.3%), and 15 (33.3%) of the tested 45 pastrami slices samples were positive for *E. faecalis*, *E. faecium*, and mixed contamination with both *E. faecalis* & *E. faecium*, respectively (Fig. 2).

Various prevalence rates of *Enterococcus* spp. have been reported in different food products tested across many countries. The prevalence rate of *Enterococcus* determined in the present study is comparable to that of Aslam et al., who found a prevalence rate of 96.3% (129/134) for *Enterococcus* in retail beef samples tested in Canada^25^. Conversely, other researchers reported lower *Enterococcus* contamination levels. For instance, Klibi et al. indicated that 80% (84/105) of fresh meat samples, including beef, mutton, and poultry, collected from Tunisian markets, were contaminated with *Enterococcus* spp.^26^, while Różańska et al. demonstrated that 61.4% (35/57) of beef meat samples in Poland were positive for enterococci^27^. Moreover, *Enterococcus* spp. were detected in raw meat and meat products at lower prevalence rates of 60% (18/30) in Brazil^28^, 60% (15/25) in Egypt^29^, 56.7% (34/60) in Canada^30^, as well as 45% (45/100)^31^, and 30.8% (70/227)^32^ in Italy.

### Distribution of *Enterococcus* isolates among shawarma sandwiches, Hawawshi sandwiches, and pastrami slices

Of the 270 molecularly identified *Enterococcus* isolates, 99 (36.7%) were identified as *E*. *faecalis*, and 171 (63.3%) were *E*. *faecium*. The distribution of *E. faecalis* and *E. faecium* isolates among RTE meat product samples indicated that 33 (36.7%) and 57 (63.3%) of the isolates recovered from Shawarma sandwiches; 39 (43.3%) and 51 (56.7%) of the isolates recovered from Hawawshi samples; and 27 (30%) and 63 (70%) of the isolates recovered from pastrami samples were positive for *E. faecalis*, *E. faecium*, respectively (Table 1).

**Table 1:** Distribution of *Enterococcus* isolates (*E*. *faecalis* *n* = 99; *E*. *faecium* *n* = 171) recovered from ready-to-eat shawarma, Hawawshi, and pastrami samples, and the associated virulence and antimicrobial resistance gene profiles

*Enterococcus spp*.	RTE meat products	No. of isolates	Virulence genes			Antimicrobial resistance gene		
			*sodA*	*gelE*	*ace*	*vanA*	*vanB*	*vanA+vanB*
*E*. *faecalis*	Shawarma sandwiches	33	33 (100%)	33 (100%)	27 (81.8%)	22 (66.7%)	0 (0%)	5 (15.2%)
	Hawawshi	39	39 (100%)	39 (100%)	39 (100%)	29 (74.4%)	0 (0%)	10 (25.6%)
	Pastrami slices	27	27 (100%)	27 (100%)	25 (92.6%)	20 (74.1%)	1 (3.7%)	6 (22.2%)
	All RTE products	99	99 (100%)^a^	99 (100%)^a^	91 (91.9%)^a^	71 (71.7%)^a^	1 (1.0%)^a^	21 (21.2%)^a^
*E*. *faecium*	Shawarma sandwiches	57	57 (100%)	34 (59.7%)	27 (47.37%)	37 (64.9%)	1 (1.8%)	8 (14.0%)
	Hawawshi	51	51 (100%)	21 (41.2%)	25 (49.02%)	38 (74.5%)	0 (2.0%)	3 (5.9%)
	Pastrami slices	63	63 (100%)	38 (60.3%)	33 (52.38%)	38 (60.3%)	3 (4.8%)	10 (15.9%)
	All RTE products	171	171 (100%)^a^	93 (54.4%)^b^	85 (49.7%)^b^	113 (66.1%)^a^	4 (2.3%)^b^	21 (12.3%)^b^
Overall number of ***Enterococcus*** spp. isolates		270	270 (100%)	192 (71.1%)	176 (65.2%)	184 (68.1%)	5 (1.9%)	42 (15.6%)

*sodA* superoxide dismutase gene, *gelE* gelatinase gene, *ace* collagen-binding protein gene; *vanA* and *vanB*: vancomycin resistance genes. Values with different superscript letters (a-b) indicate a statistically significant difference (*P* < 0.05) in virulence and resistance genes prevalence between *E*. *faecalis* and *E*. *faecium* for each antibiotic tested.

The current findings demonstrated that *E*. *faecium* was the most predominant species, followed by *E*. *faecalis*, in the RTE meat products tested. These results were consistent with those reported by Valenzuela et al., who revealed that *E*. *faecium* was the most dominant species (64%, 16/25), followed by *E*. *faecalis* (36%, 9/25), among *Enterococcus* isolates collected from foods of animal origin in Serbia^33^. Likewise, *E*. *faecium* was the most common species, with a prevalence of 61.7% (37/60), while *E*. *faecalis* was detected in 20% (12/60) among the isolates obtained from meat products in Brazil^28^.

The prevalence rate of 63.3% (171/270) for *E*. *faecium* among *Enterococcus* isolates in the present study is higher than those reported worldwide. A lower prevalence rate of 44.8% (13/29) was reported for *E*. *faecium* among enterococci isolates recovered from meat and fermented dry sausage in Canada^30^. Lower prevalence rates of *E*. *faecium* were reported in Italy, with proportions of 35.6% (16/45)^31^ and 35.7% (25/70)^32^ among enterococcal isolates from meat samples. Similarly, *E*. *faecium* showed low prevalence in meat-derived *Enterococcus* isolates from other countries, including Botswana (28.6%, 77/269)^34^, Tunisia (25%, 30/119)^26^, Portugal (24.2%, 44/182)^35^, Slovenia (15.5%, 11/71)^36^, and Canada (2.3%, 3/129)^25^.

The prevalence rate of 36.7% (99/270) determined for *E*. *faecalis* among the *Enterococcus* isolates in the present study is comparable to that reported in Italy, where *E*. *faecalis* accounted for 44.3% (31/70) of enterococcal isolates recovered from beef^32^. A slightly lower prevalence of 33.3% (15/45) was reported for *E*. *faecalis* among *Enterococcus* isolates recovered from meat samples in Italy^31^. On the other hand, *E*. *faecalis* was identified with higher prevalence rates among *Enterococcus* isolates from fresh beef tested in many countries. For instance, high rates of 84.5% (60/71) in Slovenia^36^, 73% (94/129) in Canada^25^, 57.2% (154/269) in Botswana^34^, and 44.3% (31/70) in Italy^32^. Additionally, prevalence rates of 51.7% (15/29) in Canada^30^ and 41.8% (76/182) in Portugal^35^ were reported for *E*. *faecalis* among the *Enterococcus* isolates from fermented meat products.

Notably, all *Enterococcus* isolates recovered from the ready-to-eat (RTE) bovine meat products in the present study were identified as *E. faecium* or *E. faecalis*. Other *Enterococcus* species—such as *E. casseliflavus*, *E. gallinarum*, *E. durans*, and *E. hirae*—were not detected among the isolates. Their absence may be attributable to the fact that these species are more commonly associated with other meat matrices, particularly poultry and pork meat^37,38^.

### Prevalence of virulence and resistance genes among *Enterococcus* species isolated from RTE meat products

**Fig. 3:**
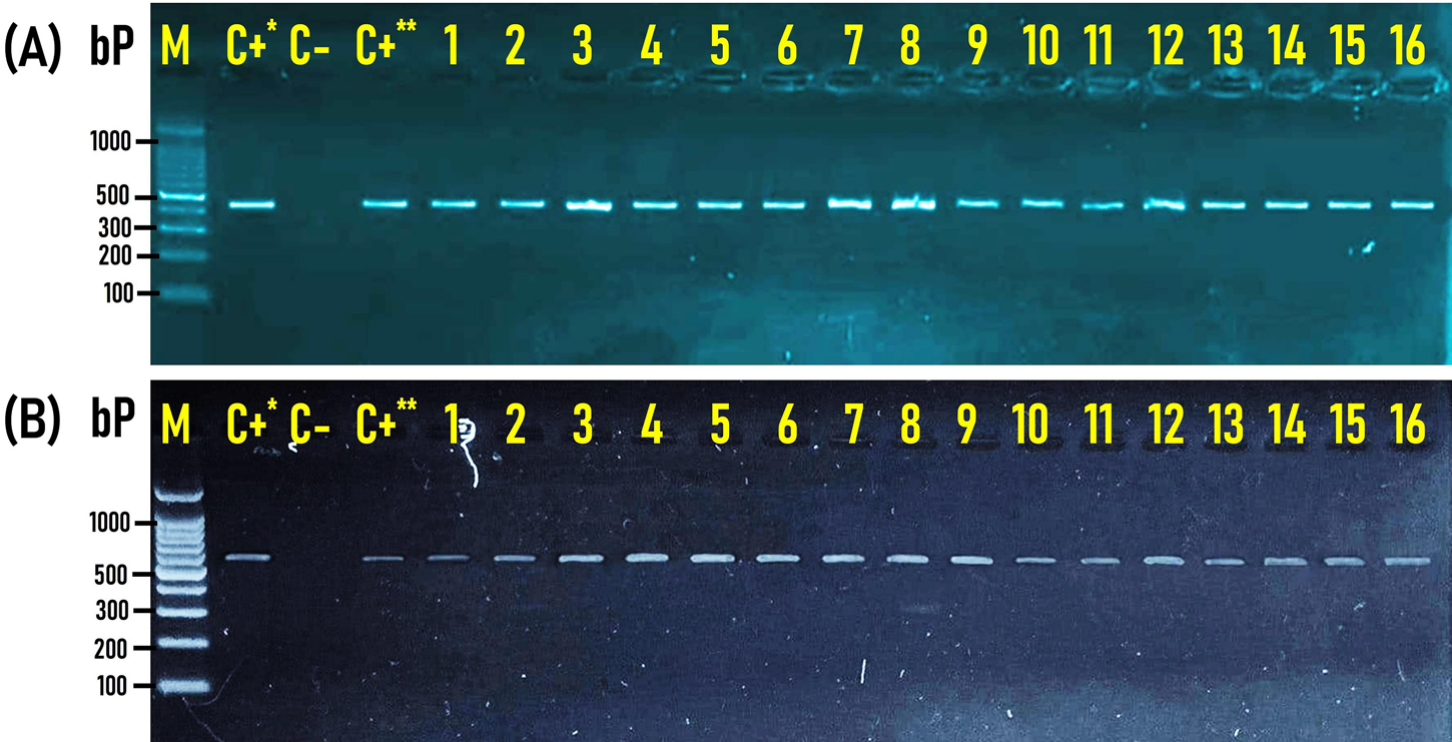
Genetic identification of gelE and ace virulence genes in *Enterococcus* isolates. Representative Agarose Gel Electrophoresis for PCR demonstrated the amplified bands of the virulent genes *gelE* (**A**) and *ace* (**B**) in *Enterococcus faecalis* (Lanes 1-8) and *Enterococcus faecium* (Lanes 9-16) isolates recovered from RTE meat products. The amplified bands were detected at the expected molecular size of 419 bp specific for the *gelE* gene (A) and 616 bp for the *ace* gene (**B**). M: DNA marker (100 bp gene ladder). C+*: Control positive for *E*. *faecalis* ATCC 29212 strain; C+**: Control positive for *E*. *faecium* ATCC 19434 strain; C–: Control negative (*Escherichia coli* K-12 DH5α). Eight microliters of the PCR product were separated by electrophoresis on a 1.5% agarose gel and visualized under UV light.

Enterococci hold potential virulence factors, estimating their severity in food, such as aggregation substance (*aS*), collagen-binding protein (*ace*), cell wall adhesion (*efaA*), enterococcal surface protein (*esp*), cytolysin (*cyl*), gelatinase (*gelE*), and hyaluronidase (*hyl*) genes^15^. In addition to the *sodA* gene, which is detected in all of the enterococci isolates in the present study and can function as both a marker gene and a virulence gene, PCR was also employed to evaluate the virulence of *Enterococcus* isolates by targeting the *gelE*, which was detected at the proper molecular size of 419 bp (Fig. 3A) and *ace* genes which was detected at the appropriate size of 616 bp (Fig. 3B).

Gelatinase (*gelE*) is a key virulence factor, playing a crucial role in protecting the bacterium from host immune responses and contributing to resistance against antimicrobial peptides and the host’s innate immune system^11,39^. It is an extracellular, zinc-dependent metalloendopeptidase which is capable of hydrolyzing host proteins, gelatin, elastin, collagen, hemoglobin, and other bioactive peptides, as well as enhancing biofilm-forming ability of *Enterococcus* strains, underscoring the significance of the *gelE* gene in virulence and host colonization^12^. The collagen-binding protein (*ace*) is recognized as a significant virulence factor in *Enterococcus* spp. It encodes a surface-fixed protein that facilitates bacterial adhesion to collagen, a major structural component of the extracellular matrix in human tissues, especially in damaged or inflamed areas, thereby promoting the persistence of infection. Moreover, the *ace* gene has a role in the formation of biofilms, which enhance bacterial survival by increasing resistance to the host immune responses and antimicrobial agents, which is a key factor in the pathogenesis of nosocomial infections. Its presence is strongly linked to serious infections such as endocarditis, urinary tract infections (UTIs), and bacteremia. Additionally, the protein encoded by *ace* may play a role in immune evasion by attaching the bacteria to host tissues, thereby reducing phagocytosis and immune recognition^13,15^.

**Fig. 4:**
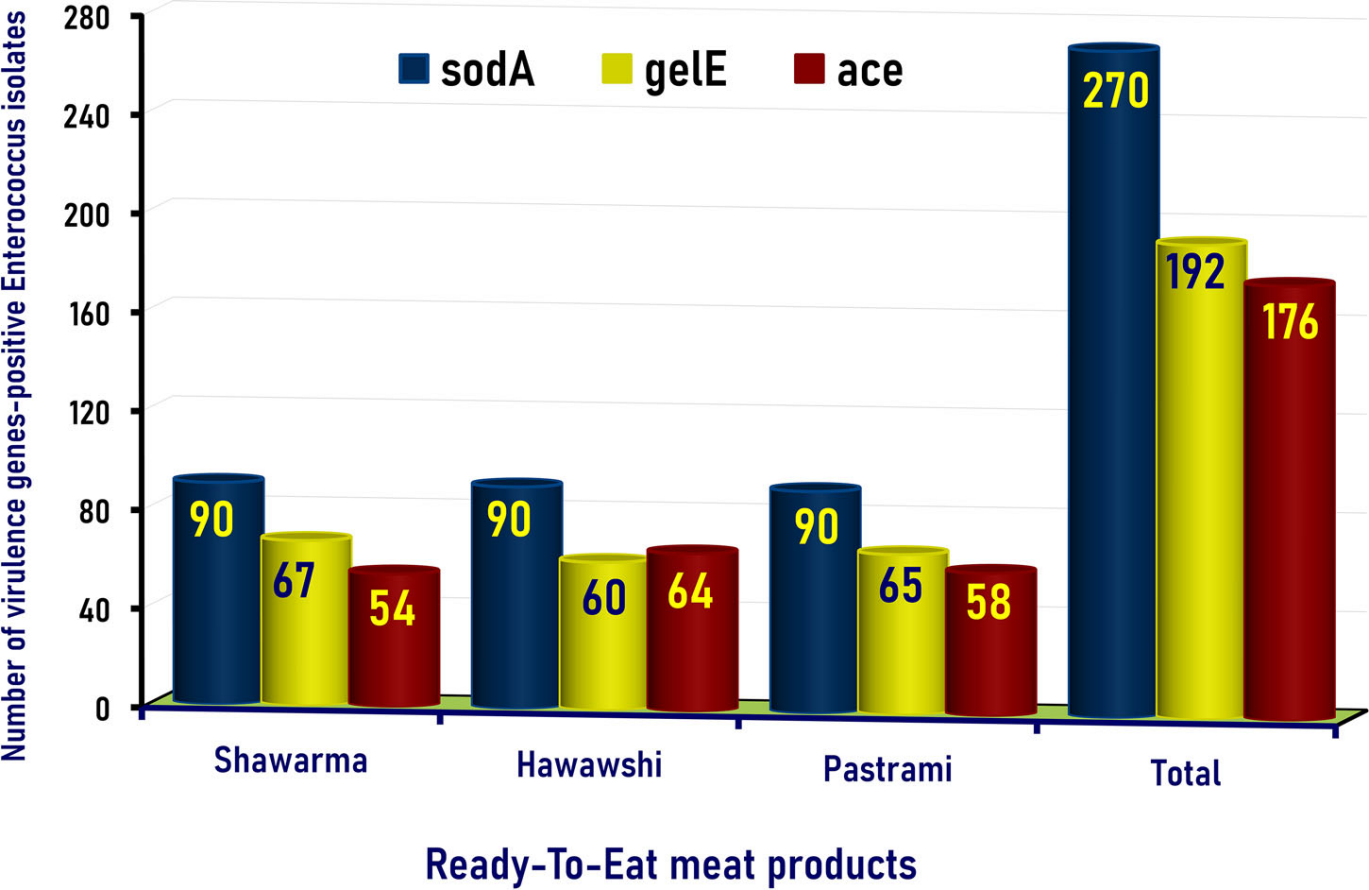
Prevalence and distribution of the virulent genes (*sodA*, *gelE*, and *ace*) among *Enterococcus spp*. isolates (*n* = 270) recovered from the examined RTE meat products.

The distribution of *sodA*, *gelE*, and *ace* genes among the 270 *Enterococcus* isolates is displayed in Fig. 4. The distribution of virulence and antimicrobial resistance genes in *E*. *faecalis* (n = 99) and *E*. *faecium* (n = 171) isolates recovered from RTE samples tested is shown in Table 1. The *sodA* gene that differentiates *Enterococcus* species into *E. faecalis* and *E. faecium* existed in all of the 270 *Enterococcus* isolates recovered from RTE meat products, while *gelE* was the predominant virulence gene among the isolates recovered from shawarma sandwiches, Hawawshi, and pastrami slices with an incidence of 74.4% (67/90), 66.7% (60/90), and 72.2% (65/90), respectively, and an overall existence of 71.1% (192/270) (Fig. 4; Table 1). On the other hand, the *ace* gene was detected in *Enterococcus* isolates recovered from the corresponding RTE meat products with an incidence of 60% (54/90), 71.1% (64/90), and 64.4% (58/90), respectively, and an overall existence of 65.2% (176/270) (Fig. 4; Table 1).

Interestingly, 100% (99/99) and 91.9% (91/99) of *E*. *faecalis* isolates were positive for *gelE* and *ace* genes, respectively, while only 54.4% (93/171) and 49.7% (85/171) of *E. faecium* isolates were positive for the *gelE* and *ace* genes, respectively. Such a difference in virulence gene distribution between the two species was statistically significant (*P* < 0.01). (Table 1). The higher prevalence of virulence factors (*gelE* and *ace* genes) in *E. faecalis* renders it more virulent than *E. faecium*. Likewise, prevalence rates of 81.6% (40/49) and only 20% (6/30) were determined for the *gelE* gene in *E*. *faecalis* and *E*. *faecium* isolates obtained from meat samples from Tunisian markets, respectively^26^. Additionally, the *gelE* gene was detected in 75% (75/100) of *E*. *faecalis* isolates obtained from beef meat samples in Turkey^40^. Similar to our findings, Gomes et al. found that 67.3% (33/49) and 24.5% (12/49) of the *E*nterococcus isolates obtained from meat products in Brazil were positive for the *gelE* and ace genes, respectively, with 100% (12/12) and 83.3% (10/12) of *E*. *faecalis* isolates harboring the *gelE* and *ace* genes, respectively gene and only 56.7% (21/37) and 5.4% (2/37) of *E*. *faecium* isolates harboring the *gelE* and *ace* gene, respectively^28^.

The *gelE* and *ace* genes were also detected in 80% (12/15) of *E*. *faecalis* and only 38.5% (5/13) and 0% (0/13) of *E*. *faecium* isolates recovered from raw and fermented meat in Canada, respectively^41^. Likewise, high prevalence rates of 65.8% (79/121) and 76.7% (92/120) were reported for *gelE* and *ace* genes, respectively, in *E*. *faecalis*, although the corresponding genes were not detected (0%; 0/21) in *E*. *faecium* isolates recovered from retail red meat in Slovenia^36^. Conversely, existence rates of 71.4% (5/7) and 42.9% (3/7) were determined for *gelE* and *ace* genes in *E*. *faecalis*, versus a higher existence rate of 100% (2/2) for both genes in *E*. *faecium* isolates recovered from meat and dairy products tested in Egypt^29^. The *gelE* and *ace* genes were found at prevalence rates of 36% (9/25) and 0% (0/25) in *Enterococcus* isolates recovered from dairy and meat products in Serbia, with a higher frequency of *gelE* gene in *E*. *faecalis* (44.4%; 4/9) than in *E*. *faecium* (31.3%; 5/16)^33^. Lower existence rates of 35% (7/20) and 5% (1/20) were also reported for *gelE* and *ace* genes, respectively, in *E*. *faecalis* strains isolated from dry fermented sausages in Portugal^42^.

Analysis of vancomycin resistance genes revealed that the *vanA* gene was prevalent in both *E. faecalis* and *E. faecium* isolates, with no significant interspecies difference. In contrast, the *vanB* gene and the co-occurrence of *vanA*/*vanB* differed significantly between the two species (*P* < 0.05 and P < 0.01, respectively), with higher detection of *vanB* among *E. faecium* isolates and a higher frequency of *vanA*/*vanB* coexistence among *E. faecalis* isolates (Table 1).

### Antimicrobial resistance profile of *Enterococcus* isolates (n = 270) from RTE meat products

The global increase in antibiotic resistance poses a serious threat by reducing the effectiveness of commonly used antibiotics in treating widespread bacterial infections. The 2022 Global Antimicrobial Resistance and Use Surveillance System (GLASS) report is alarming about resistance rates among prevalent bacterial pathogens^43^. The Antimicrobial resistance profile of all verified *E*. *faecalis* and *E*. *faecium* isolates (*n* = 270) toward 17 antibiotics related to 12 antimicrobial classes tested is shown in Table 2.

**Table 2:** The antimicrobial resistance profile of enterococci isolates from RTE meat products

*Enterococcus* sources	Isolates	Number and (%) of the isolates resistant to the 14 tested antimicrobials													
		C	CIP	SXT	LZD	LEV	AZM	IPM	RD	VA	NA	CN	P	AMX	TE
Shawarma sandwiches	*E. faecalis* (*n* = 33)	0 (0%)	20 (60.6%)	19 (57.6%)	0 (0%)	7 (21.2%)	21 (63.6%)	33 (100%)	26 (78.8%)	27 (81.8%)	7 (21.2%)	14 (42.4%)	33 (100%)	13 (39.4%)	19 (57.6%)
	*E*. *faecium* (n = 57)	0 (0%)	46 (80.7%)	36 (63.2%)	35 (61.4%)	34 (59.6%)	46 (80.7%)	*NA	46 (80.7%)	46 (80.7%)	57 (100%)	22 (38.6%)	57 (100%)	23 (40.6%)	34 (59.6%)
	Overall (*n* = 90)	0 (0%)	66 (73.3%)	55 (61.1%)	35 (38.9%)	41 (45.6%)	67 (74.4%)	33 (100%)	72 (80%)	73 (81.1%)	64 (71.1%)	36 (40%)	90 (100%)	36 (40%)	53 (58.9%)
Hawawshi	*E. faecalis* (n = 39)	0 (0%)	16 (41%)	31 (79.5%)	16 (41%)	23 (59%)	31 (79.5%)	39 (100%)	31 (79.5%)	39 (100%)	8 (20.5%)	15 (38.5%)	39 (100%)	8 (20.5%)	39 (100%)
	*E*. *faecium* (*n* = 51)	0 (0%)	20 (39.2%)	41 (80.4%)	0 (0%)	21 (41.2%)	41 (80.4%)	*NA	41 (80.4%)	41 (80.4%)	10 (19.6%)	10 (19.6%)	51 (100%)	21 (41.2%)	41 (80.4%)
	Overall (*n* = 90)	0 (0%)	36 (40%)	72 (80%)	16 (17.8%)	44 (48.9%)	72 (80%)	39 (100%)	72 (80%)	80 (88.9%)	18 (20%)	25 (27.8%)	90 (100%)	30 (33.3%)	80 (88.9%)
Pastrami slices	*E. faecalis* (*n* = 27)	0 (0%)	0 (0%)	21 (77.8%)	21 (77.8%)	0 (0%)	5 (18.5%)	27 (100%)	27 (100%)	27 (100%)	0 (0%)	6 (22.2%)	27 (100%)	11 (40.7%)	10 (37%)
	*E*. *faecium* (*n* = 63)	0 (0%)	25 (39.7%)	38 (60.3%)	13 (20.6%)	38 (60.3%)	37 (58.7%)	*NA	63 (100%)	51 (81%)	25 (39.7%)	37 (58.7%)	63 (100%)	37 (58.7%)	37 (58.7%)
	Overall (*n* = 90)	0 (0%)	25 (27.8%)	59 (65.6%)	34 (37.8%)	38 (42.2%)	42 (46.7%)	27 (100%)	90 (100%)	78 (86.7%)	25 (27.8%)	43 (47.8%)	90 (100%)	48 (53.3%)	47 (52.2%)
Overall isolates	*E*. *faecalis* (*n* = 99)	0 (0%)	36 (36.3%)^a^	71 (71.7%)^a^	37 (37.3%)^a^	30 (30.3%)^a^	57 (57.6%)^a^	99 (100%)	84 (84.4%)^a^	93 (93.9%)^a^	15 (15.2%)^a^	35 (35.4%)^a^	99 (100%)^a^	32 (32.3%)^a^	68 (68.7%)^a^
	*E*. *faecium* (*n* = 171)	0 (0%)	91 (53.2%)^b^	115 (67.3%)^a^	48 (28.1%)^a^	93 (54.4%)^b^	124 (72.5%)^b^	*NA	150 (87.7%)^a^	138 (80.7%)^a^	92 (53.8%)^b^	69 (40.4%)^a^	171 (100%)^a^	81 (47.4%)^b^	122 (71.3%)^a^
Overall enterococci resistance (*n* = 270)		0 (0%)	127 (47%)	186 (68.9%)	85 (31.5%)	123 (45.6%)	181 (67%)	99 (100%)	234 (86.7%)	231 (85.6%)	107 (39.6%)	104 (38.5%)	270 (100%)	113 (41.9%)	190 (70.4%)

*C* Chloramphenicol, *CIP* Ciprofloxacin, *SXT* Trimethoprim/Sulfamethoxazole, *LZD* Linezolid, *LEV* Levofloxacin, *AZM* Azithromycin, *IMP* Imipenem, *RD* Rifampin, VA Vancomycin, NA: Nalidixic acid, CN Gentamicin (Susceptibility testing for *Enterococcus* spp. was performed exclusively to detect high-level resistance using high-content disks); *P* Penicillin, *Amx* Amoxicillin, TE Tetracycline. *NA Not applicable. Values with different superscript letters indicate a statistically significant difference (*P* < 0.05) in resistance rates between *E*. *faecalis* and *E*. *faecium* for each antibiotic tested.

In the current study, *Enterococcus* isolates showed absolute resistance to several antibiotics related to different classes linked to β-lactams. Interestingly, 100% of the isolates were resistant to at least one β-lactam antibiotic. All (100%) *E*. *faecalis* (99/99) and *E*. *faecium* (171/171) isolates showed resistance to ampicillin (Table 2). Lower resistance rates ranging from 0% to 10.7% were reported against penicillin and ampicillin among *E*. *faecium* and *E*. *faecalis* isolates recovered from different meat products tested in various countries, including Canada^25,30^, Poland^27^, Portugal^42^, and Turkey^40^. Absolute resistance rate of 100% (99/99) was also reported for *E. faecalis* isolates towards imipenem (Table 2). A markedly lower resistance rate of 22.2% (10/45) was determined by *Enterococcus* isolates recovered from meat samples in Italy^31^. The results also indicated that 86.7% (234/270) of *E*. *faecalis* and *E*. *faecium* were resistant to rifampin (Table 2). A lower resistance rate of 76% (19/25) was recorded towards rifampin among *Enterococcus* isolates obtained from foods of animal origin in Serbia^33^. Likewise, lower resistance rates of 60% (109/182)^35^ and 20% (9/45)^31^ were reported for enterococcal isolates recovered from meat products tested in Portugal and Italy, respectively.

Vancomycin has long been considered a drug of choice due to its efficiency in treating multidrug-resistant infectious agents. This study revealed high resistance rates of 93.9% (93/99) and 80.7% (138/171) towards vancomycin among *E*. *faecalis* and *E*. *faecium* isolates, respectively, with a total resistance rate of 85.6% (231/270) for both *E*. *faecalis* and *E*. *faecium* isolates (Table 2). An approximately similar resistance rate of 75% (12/16) against vancomycin was noticed for *E. faecium* isolated from meat products by Pavia et al. in Italy, although they reported a lower resistance rate of 40% (6/15) for *E. faecalis*^31^. Likewise, 75% (24/32) of *E*. *faecalis* and *E*. *faecium* isolates from the milk of sheep and goats with subclinical mastitis in Qena, Egypt, were resistant to vancomycin^44^. Ribeiro et al., in another hand, reported that only 45% (9/20) of *E*. *faecalis* were resistant to vancomycin^42^. On the contrary, *E*. *faecalis* and *E*. *faecium* isolates obtained from retail meat in Canada^25,30^ and Slovenia^36^ showed no resistance against vancomycin.

Tetracycline is one of the most commonly employed antibiotics in human and veterinary infections due to its low cost and widespread availability^45^. In the present study, *E*. *faecalis* isolates exhibited a resistance rate of 68.7% (68/99) to tetracycline (Table 2). Likewise, a resistance rate of 67.3% (33/49) was recorded against tetracycline among *E*. *faecalis* in Tunisia^26^, while higher resistance rates of 74.3% (26/35)^27^ and 73.3% (11/15)^30^ towards tetracycline were reported for *E. faecalis* isolates obtained from meat samples examined in Poland and Canada, respectively. Conversely, lower resistance rates of 60% (12/20) in Portugal^42^, 53% (53/100) in Turkey^40^, 29.2% (35/120) in Slovenia^36^, 14.9% (14/94) in Canada^25^, and 5.8% (9/154) in Botswana^34^ were recorded by *E*. *faecalis* isolates recovered from meat samples towards tetracycline. Moreover, 71.3% (122/171) of *E*. *faecium* isolates analyzed in the present study were resistant to tetracycline (Table 2). The high resistance rates to this antibiotic in our study may be attributed to its abuse in livestock, which can easily promote the horizontal transfer of *tet* resistance genes from animals to humans via the gut microbiota, particularly within *Enterococcus* species^45^. On the contrary, lower resistance rates of 53.8% (7/13)^30^, 36.7% (11/30)^26^, 6.5% (5/77)^34^, 4.8% (1/21)^36^, and 0% (0/94)^25^ were reported for *E*. *faecium* isolates against tetracycline.

In the present study, 68.9% (186/270) of enterococci isolates showed resistance to trimethoprim/sulfamethoxazole (Table 2). Conversely, a markedly low resistance rate of only 7% (7/100) toward trimethoprim/sulfamethoxazole was reported for *E*. *faecalis* isolates recovered from minced beef samples from butcher shops and supermarkets in Turkey^40^. On the other 67% (181/270) of *Enterococcus* isolates tested in the present study were resistant to azithromycin (Table 2). The demonstrated resistance of enterococci to azithromycin arises from the presence of multiple resistance genes that block macrolide binding to the ribosome, combined with ribosomal alterations, specifically methylation of 16S rRNA and ribosomal proteins^46^^,47^.

Resistance to nalidixic acid, a first-generation quinolone, often heralds subsequent fluoroquinolone resistance and is associated with a higher likelihood of developing resistance to advanced generations (ciprofloxacin and levofloxacin)^48^. *Enterococcus* isolates exhibited resistance rates of 47% (127/270), 45.6% (123/270), and 39.6% (107/270) towards ciprofloxacin, levofloxacin, and nalidixic acid, respectively (Table 2). Higher resistance rates of 72% (18/25) and 56% (14/25) were determined for *E*. *faecalis* and *E*. *faecium* recovered from food of animal origin in Serbia against ciprofloxacin and levofloxacin, respectively^33^. On the other hand, lower resistance rates of 12% (12/100) in Turkey^40^, 10.3% (3/29) in Canada^30^, 1.4% (2/141) in Slovenia^36^, 0% (0/35) in Poland^27^, and 0% (0/94) in Canada^34^ were demonstrated against ciprofloxacin for enterococcal isolates recovered from meat samples. In *Enterococcus* spp., the resistance to quinolones is predominantly caused by chromosomal mutations within the quinolone resistance-determining regions (QRDRs) of the DNA gyrase (*gyrA*) and topoisomerase IV (parC) genes. These mutations alter the quinolone binding ability of the enzyme, thereby reducing the drug affinity^49^.

*Enterococcus* species, particularly *E*. *faecalis* and *E*. *faecium*, have demonstrated a high level of resistance to gentamicin, an aminoglycoside antibiotic commonly used in combination therapy for serious enterococcal infections such as endocarditis^50^. Aminoglycoside susceptibility testing for *Enterococcus* spp. was performed exclusively to detect high-level resistance using high-content disks. Notably, 35.4% (35/99) of *E*. *faecalis* isolates were resistant to gentamicin (Table 2). A higher resistance rate (65%; 13/20) against gentamicin was demonstrated by *E*. *faecalis* isolates recovered from dry fermented sausage^42^. While lower resistance rates of 6.7% (1/15)^30^, 1.7% (2/120)^36^, and 0%^25–27^ were demonstrated by *E*. *faecalis* isolates from different meat samples against gentamicin. On the other hand, 40.4% (69/171) of *E*. *faecium* exhibited resistance to gentamicin (Table 2). A much lower resistance rate of 3.3% (1/30) was estimated among *E*. *faecium* isolates from meat in Tunisia^26^, while none of the *E*. *Faecium* isolates in Canada^25,39^ or in Slovenia^36^ showed resistance to gentamicin.

All (270/270) *E*. *faecalis* and *E*. *faecium* showed no resistance to chloramphenicol (Table 2). This result is comparable with that of Golob et al., who found that all *Enterococcus* isolates obtained from red meat were susceptible to chloramphenicol^36^. On the contrary, higher resistance rates of 28.6% (10/35)^27^ and 10.3% (3/29)^30^ were reported for enterococci isolates towards chloramphenicol.

Amoxicillin is the drug of choice for the treatment of enterococcal urinary tract infections, while vancomycin is the second-line therapy. Alternative therapies include linezolid; however, it would ideally be reserved for more serious infections to preserve activity. Interestingly, the present results demonstrated that 32.3% (32/99) of *E*. *faecalis* and 47.4% (81/171) of *E*. *faecium* were resistant to amoxicillin, with an overall resistance rate of 41.9% (113/270) for the enterococci isolates (Table 2). Such a high resistance rate is considered alarming, as it reflects a substantial reduction in the effectiveness of first-line β-lactam therapy and indicates significant selective pressure within the food production environment. In comparison with our findings, previous studies on enterococci from food reported much lower amoxicillin resistance rates. For instance, only one from 312 *Enterococcus* species (0.32%), definitely *E*. *faecium*, isolated from retail food of animal origin in Italy, was resistant to Amoxicillin-clavulanic acid^32^, while 19% (38/200) of the enterococci recovered from retail red meat and poultry preparations in Spain were resistant to Ampicillin^51^. On the other hand, many reports on enterococci recovered from environmental and human clinical samples in many developing countries showed higher resistance rates comparable to our findings. In Egypt, 80.7% (46/54) of *E*. *faecium* and 45.7% (32/70) of *E*. *faecalis*, with an overall enterococcal resistance of 61.4% (78/127), isolated from urinary tract infections in Zagazig University hospitals, Egypt, were resistant to amoxicillin^52^. Likewise, 100% (26/26) of tested *E. faecalis* isolates from surgical wound infection from Egyptian patients with hospital-acquired infections were resistant to ampicillin and 18 (69.2%) to amoxicillin-clavulanic^53^. In Nigeria, 63.9% (27/36) of *E*. *faecium* and 62.1% (19/29) of *E*. *faecalis* from clinical samples of humans showed high resistance to amoxicillin-clavulanate^54^. Similarly, 95.7% (22/23) of *Enterococcus* isolates, including 18 *E. faecalis* and 5 *E. faecium*, identified from environmental, animal, and clinical samples from Nigeria, demonstrated high resistance levels for amoxicillin–clavulanic acid^55^. Furthermore, among 24 isolates of human clinical samples in Ethiopia, 16 isolates (66.7%) were resistant to ampicillin, penicillin, and amoxicillin/clavulanic acid^56^.

Linezolid is regarded as a last-line treatment option for severe infections caused by Gram-positive bacteria that are resistant to other antibiotics, including vancomycin-resistant *Enterococcus* (VRE) and methicillin-resistant *Staphylococcus* species^57^. Among 270 enterococcal isolates of the present study, 85 (31.5%) exhibited resistance to linezolid (Table 2). A lower resistance rate of 11.4% (4/35) was reported for *E*. *faecalis* toward linezolid^27^, while all isolates tested in Canada^25^ and Slovenia^36^ were susceptible to linezolid. In contrast higher resistance rate of 70.3% was reported by enterococci isolates from food of animal origin against linezolid^44^.

If you want it slightly more formal or more compact (e.g., table-focused wording), I can adjust it further. The high prevalence of resistance to amoxicillin and vancomycin in this study warrants particular emphasis due to their importance in food safety and public health. Notably, of the 270 *Enterococcus* isolates tested, 113 (41.9%) were resistant to amoxicillin and 231 (85.6%) to vancomycin, indicating a substantial presence of VRE in the analyzed ready-to-eat foods. Furthermore, 85 isolates (31.5%) exhibited linezolid resistance, a concerning finding given that linezolid is one of the last-resort agents for treating VRE infections. These findings underscore the clinical relevance of the observed resistance patterns and highlight the need for strengthened surveillance, antimicrobial stewardship, and improved hygiene practices to limit the dissemination of multidrug-resistant *Enterococcus* through the food chain.

Comparative analysis of antimicrobial resistance profiles demonstrated that *E*. *faecium* exhibited significantly higher resistance rates to ciprofloxacin, levofloxacin, azithromycin, nalidixic acid, and amoxicillin than *E*. *faecalis* (P < 0.01), whereas no significant interspecies differences were observed for the remaining nine antibiotics tested (Table 2).

### Multiple antibiotic resistance (MAR) index and classification of *Enterococcus* isolates based on their antibiotic resistance profiles

Antibiotic resistance profiles and multiple-antibiotic resistance index (MAR) of *Enterococcus* spp. isolated from shawarma sandwiches, Hawawshi, and pastrami slices are shown (Table 3). No pan-drug-resistant (PDR) strains were identified among the enterococci analyzed from the non-reheated RET meat samples examined in the present study. Nonetheless, 13.7% (37/270) *Enterococcus* isolates, including 15 *E*. *faecalis* (8 from Hawawshi and 7 from shawarma sandwiches) and 22 *E*. *faecium* (12 from shawarma sandwiches and 10 from Hawawshi), were categorized as extensively drug-resistant, with MAR index ranging between 0.857 and 0.929 (Table 3). Furthermore, 86.3% (233/270) of *Enterococcus* isolates were classified as multidrug-resistant (MDR) with an MAR index ranging between 0.286 and 0.786 (Table 3). Unexpectedly, none of the isolates were classified as low-drug resistant, and all (100%, 270/270) of the enterococcal isolates were resistant to at least four antibiotics, while 96.3% (260/270) of the *Enterococcus* isolates were resistant to at least 6 antibiotics (Table 3). Different MDR patterns among *Enterococcus* isolates collected from various meat products were recorded in other studies globally. For instance, Jahan et al.^30^ in Canada reported that 58.6% (17/29) of *E. faecalis* and *E*. *faecium* isolates exhibited resistance to 3 to 8 antibiotics. Similarly, 58.3% of *E. faecalis* and 64.3% of *E. faecium* isolates (overall, 57.5% of isolates) from dairy products marketed in Qena city, Egypt, were MDR^58^, while Klibi et al. in Tunisia declared that 24.5% (29/119) of examined enterococci were resistant to at least four antibiotics^26^. On the other hand, Yılmaz et al. in Turkey reported that 63% (63/100) of enterococci isolates were resistant to at least one antibiotic^40^, whereas Golob et al. in Slovenia revealed that 32.6% (46/141) of tested enterococci isolates were resistant to either one or two antimicrobials^36^.

**Table 3:** Classification of *Enterococci* isolates (*n* = 270) based on their antibiotic resistance profile against fourteen antibiotics tested and their multiple antibiotic resistance (MAR) index

*Enterococcus* spp.	Antimicrobial resistance profile	Sources	MAR index	Classification of strains	
				Type of resistance	No. and %
***E***. ***faecalis***	C, P, IPM, VA, RD, SXT, TE, AZM, LZD, CIP, CN, LEV, NA	Hawawshi sandwich (*n* = 8)	0.929	Extensively drug-resistant	15 (15.2%)
	C, P, IPM, VA, RD, TE, AZM, CIP, CN, LEV, NA, AMX	Shawarma sandwich (*n* = 7)	0.857		
	C, P, IPM, VA, RD, SXT, TE, AZM, CIP, LEV, AMX	Hawawshi sandwich (*n* = 8)	0.786	Multidrug-resistant	84 (84.8%)
	C, P, IPM, VA, RD, SXT, TE, AZM, CN, LEV	Hawawshi sandwich (*n* = 7)	0.714		
	C, P, IPM, VA, RD, SXT, AZM, LZD, AMX	Pastrami slices (*n* = 5)	0.642		
	C, P, IPM, VA, RD, LZD, CN, AMX	Pastrami slices (*n* = 6)	0.571		
	C, P, IPM, VA, SXT, TE, AZM, LZD	Hawawshi sandwich (*n* = 8)	0.571		
	C, P, IPM, VA, RD, SXT, TE, LZD	Pastrami slices (*n* = 5)	0.571		
	C, P, IPM, RD, SXT, TE, CIP, AMX	Shawarma sandwich (*n* = 6)	0.571		
	C, P, IPM, VA, RD, SXT, TE, LZD	Pastrami slices (*n* = 5)	0.571		
	C, P, IPM, VA, SXT, AZM, CN	Shawarma sandwich (*n* = 7)	0.500		
	C, P, IPM, VA, RD, AZM, CIP	Shawarma sandwich (*n* = 7)	0.500		
	C, P, IPM, VA, RD, SXT, TE	Shawarma sandwich (*n* = 6)	0.500		
	C, P, IPM, VA, RD, SXT	Pastrami slices (*n* = 6)	0.429		
	C, P, IPM, VA, RD, TE	Hawawshi sandwich (*n* = 8)	0.429		
**Sum 99**	**Average MAR Index for** ***E***. ***faecalis***		**0.576**		
***E. faecium***	C, P, RD, VA, AZM, TE, SXT, LEV, NA, CIP, LZD, AMX	Shawarma sandwich (*n* = 12)	0.857	Extensively drug-resistant	22 (12.87%)
	C, P, RD, VA, AZM, TE, SXT, LEV, NA, CIP, CN, AMX	Hawawshi sandwich (*n* = 10)	0.857		
	C, P, RD, VA, AZM, TE, LEV, NA, CIP, CN, AMX	Shawarma sandwich (*n* = 11)	0.786	Multidrug-resistant	149 (87.13%)
	C, P, RD, VA, AZM, LEV, NA, CIP, CN, AMX	Pastrami slices (*n* = 13)	0.714		
	C, P, RD, AZM, TE, LEV, NA, CIP, CN, AMX	Pastrami slices (*n* = 12)	0.714		
	C, P, RD, VA, TE, SXT, LEV, NA, CIP, CN	Shawarma sandwich (*n* = 11)	0.714		
	C, P, RD, VA, AZM, TE, SXT, CN, AMX	Pastrami slices (*n* = 12)	0.643		
	C, P, RD, VA, AZM, TE, SXT, LEV, AMX	Hawawshi sandwich (*n* = 11)	0.643		
	C, P, RD, VA, AZM, SXT, NA, CIP, LZD	Shawarma sandwich (n = 12)	0.643		
	C, P, RD, VA, AZM, TE, SXT	Hawawshi sandwich (*n* = 10)	0.500		
	C, P, RD, VA, TE, SXT, LEV	Pastrami slices (*n* = 13)	0.500		
	C, P, RD, VA, TE, SXT, CIP	Hawawshi sandwich (*n* = 10)	0.500		
	C, P, RD, VA, SXT, LZD	Pastrami slices (*n* = 13)	0.429		
	C, P, AZM, NA, LZD	Shawarma sandwich (*n* = 11)	0.357		
	C, P, AZM, TE	Hawawshi sandwich (*n* = 10)	0.286		
**Sum 171**	**Average MAR Index for** ***E***. ***faecium***		**0.622**		
**Sum 270**	***E***. ***faecium*** **&** ***E***. ***faecalis***		**0.604**	Extensively drug-resistant Multidrug-resistant	37 (13.7%) 233 (86.3%)

*C* Chloramphenicol, *P* Penicillin, *IMP* Imipenem, *VA* Vancomycin, *RD* Rifampin, *SXT* Trimethoprim/Sulfamethoxazole, *TE* Tetracycline, *AZM* Azithromycin, *LZD* Linezolid, *CIP* Ciprofloxacin, *CN* Gentamicin, LEV: Levofloxacin *NA* Nalidixic acid, *AMX* Amoxicillin.

It has been noticed that *E*. *faecalis* revealed a mean MAR index of 0.576, while *E*. *faecium* exhibited a mean MAR index of 0.622, with an overall average MAR index of 0.604 for all 270 enterococcus isolates tested (Table 3). Interestingly, 92.2% (249/270) of *Enterococcus* isolates exhibited a MAR index exceeding 0.4 (Table 3). A MAR index greater than 0.2 indicates misuse of antimicrobials, whereas a value exceeding 0.4 may indicate human-related fecal contamination^28^.

### Association of vancomycin resistance genes among MDR enterococci isolates

**Fig. 5:**
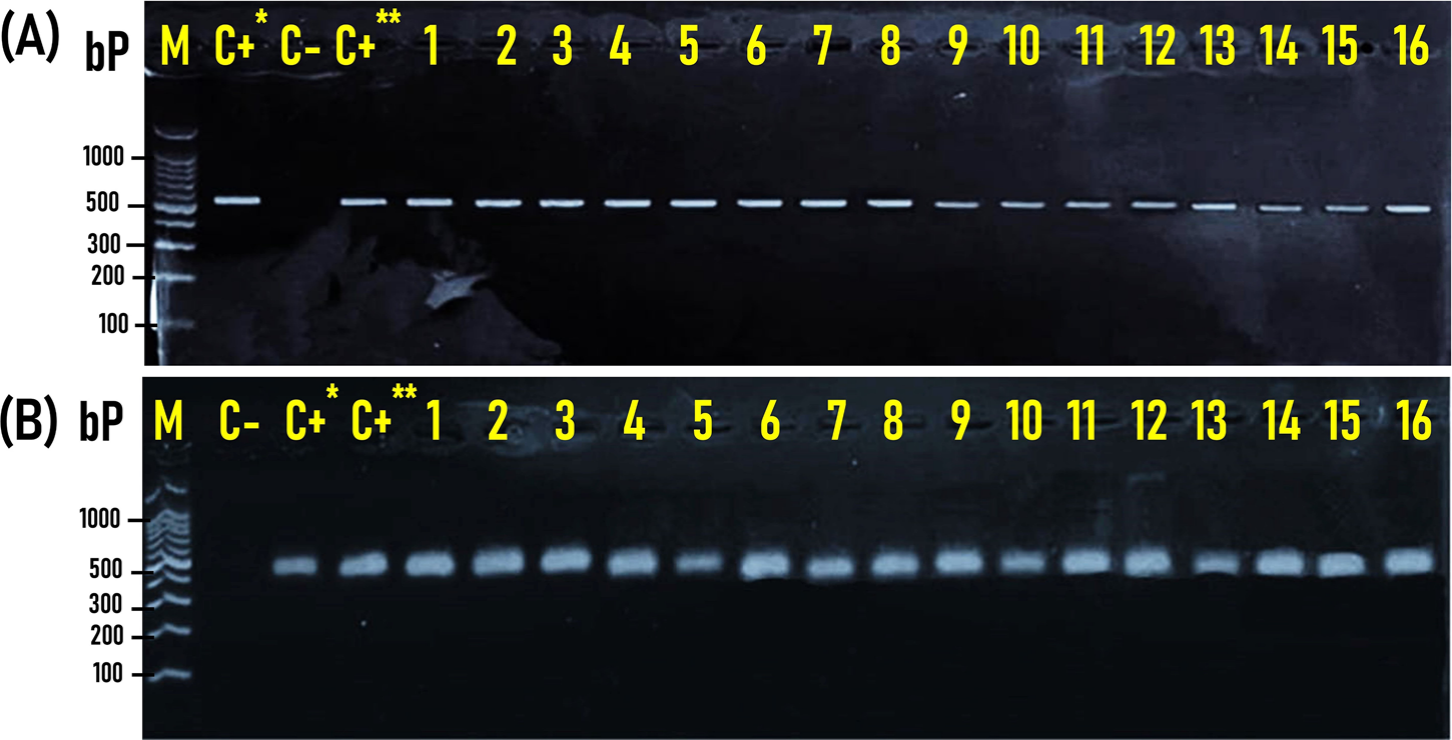
Genetic identification of vancomycin-resistant genes in *Enterococcus* isolates. Representative Agarose Gel Electrophoresis for PCR demonstrated the amplified bands of the vancomycin-resistant *vanA* (**A**) and *vanB* (**B**) in *Enterococcus faecalis* (Lanes 1–8) and *Enterococcus faecium* (Lanes 9–16) isolates recovered from RTE meat products. The amplified bands were detected at the expected molecular size of 559 bp specific for the *vanA* gene (**A**) and 467 bp for the *vanB* gene (**B**). M; DNA marker (100 bp gene ladder). C+*: Control positive for *E. faecalis* ATCC 29212 strain; C+**: Control positive for *E. faecium* ATCC 19434 strain; C–: Control negative (*Escherichia coli* K-12 DH5α). Eight microliters of the PCR product were separated by electrophoresis on a 1.5% agarose gel and visualized under UV light.

PCR targeting the vancomycin resistance genes *vanA* and *vanB* in *E*. *faecalis* (n = 99) and *E*. *faecium* (n = 171) isolates recovered from RTE meat products revealed their amplification at the expected molecular size of 559 bp (Fig. 5A) and 467 bp (Fig. 5B), respectively. The molecular (PCR) investigation verified 231 isolates as vancomycin-resistant *Enterococcus* (VRE) by detecting *vanA* and/or *vanB* genes.

**Fig. 6:**
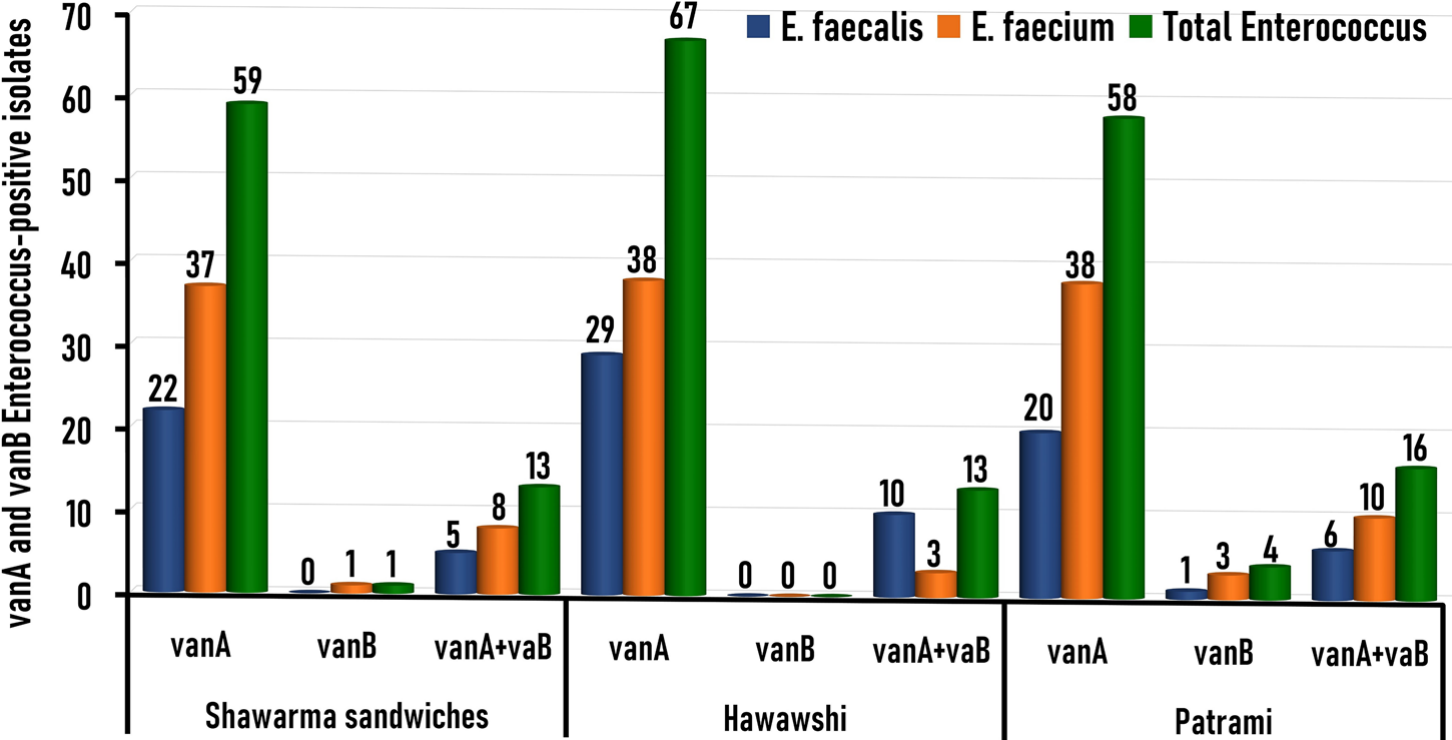
Prevalence and distribution of the vancomycin-resistant genes (*vanA a*nd *vanB*) among *Enterococcus spp*. isolates (*n* = 270) recovered from the examined RTE meat products.

Among these 231 VRE isolates, 184 (79.7%) distributed as 59, 67, and 58 isolates from Shawarma sandwiches, Hawawsshi, and pastrami slices, respectively were positive for *vanA* gene alone, 5 (2.2%) isolates distributed as 1, 0, and 4 isolates from Shawarma sandwiches, Hawawsshi, and pastrami slices, respectively were positive for *vanB* lonely, and 42 (18.2%) isolates (distributed as 13, 13, and 16 isolates from Shawarma sandwiches, Hawawsshi, and pastrami slices, respectively) harbored both *vanA* and *vanB* genes (Table 4 and Fig. 6). The *vanA* gene was the most prevalent (83.7%, 226/270), followed by the *vanB* gene (17.4%, 47/270). It is noticed that 66.7% (22/33), 74.4% (29/39), and 74.1% (20/27) of *E*. *faecalis* isolates and 64.9% (37/57), 74.5% (38/51), and 60.3% (38/63) *E*. *faecium* isolates recovered from shawarma sandwiches, Hawawsshi, and pastrami slices, respectively, were positive for *vanA* gene (Table 4 and Fig. 6). On the other hand, 15.2% (5/33), 25.6% (10/39), and 22.2% (6/27) of *E*. *faecalis* isolates and 14% (8/57), 5.9% (3/51), and 15.9% (10/63) *E*. *faecium* isolates recovered from shawarma sandwiches, Hawawsshi, and pastrami slices, respectively, were positive for *vanA+vanB* genes together (Table 4 and Fig. 6). Collectively, vancomycin resistance genes were mostly found in 96.9% (93/96) of *E*. *faecalis* isolates and 80.7% (138/171) of *E*. *faecium* isolates.

**Table 4:** Association of virulent and vancomycin-resistant genes in multidrug-resistant *Enterococcus* isolates (n = 270) isolated from RTE meat products

*Enterococcus* spp.	Source and (number)	No. of isolate	Resistance profile	Virulence gene	Resistance genes
***E***. ***faecalis*** (*n* = 99)	Shawarma sandwiches (33 isolates)	3	C, P, IPM, VA, RD, TE, AZM, CIP, CN, LEV, NA, AMX	*sodA, gelE, ace*	*VanA*, *VanB*
		4	C, P, IPM, VA, RD, TE, AZM, CIP, CN, LEV, NA, AMX	*sodA, gelE, ace*	*VanA*
		3	C, P, IPM, VA, SXT, AZM, CN, AMX	*sodA, gelE*	*VanA*
		3	C, P, IPM, RD, SXT, TE, CIP, AMX	*sodA, gelE, ace*	-
		4	C, P, IPM, VA, SXT, AZM, CN	*sodA, gelE, ace*	*VanA*
		3	C, P, IPM, RD, SXT, TE, CIP	*sodA, gelE*	-
		1	C, P, IPM, VA, RD, AZM, CIP	*sodA, gelE, ace*	*VanA*, *VanB*
		6	C, P, IPM, VA, RD, AZM, CIP	*sodA, gelE, ace*	*VanA*
		1	C, P, IPM, VA, RD, SXT, TE	*sodA, gelE, ace*	*VanA*, *VanB*
		5	C, P, IPM, VA, RD, SXT, TE	*sodA, gelE, ace*	*VanA*
	Hawawshi (39 isolates)	4	C, P, IPM, VA, RD, SXT, TE, AZM, LZD, CIP, CN, LEV, NA	*sodA, gelE, ace*	*VanA*, *VanB*
		4	C, P, IPM, VA, RD, SXT, TE, AZM, LZD, CIP, CN, LEV, NA	*sodA, gelE, ace*	*VanA*
		3	C, P, IPM, VA, RD, SXT, TE, AZM, CIP, LEV, AMX	*sodA, gelE, ace*	*VanA*, *VanB*
		5	C, P, IPM, VA, RD, SXT, TE, AZM, CIP, LEV, AMX	*sodA, gelE, ace*	*VanA*
		2	C, P, IPM, VA, RD, SXT, TE, AZM, CN, LEV	*sodA, gelE, ace*	*VanA*, *VanB*
		5	C, P, IPM, VA, RD, SXT, TE, AZM, CN, LEV	*sodA, gelE, ace*	*VanA*
		1	C, P, IPM, VA, SXT, TE, AZM, LZD	*sodA, gelE, ace*	*VanA*, *VanB*
		7	C, P, IPM, VA, SXT, TE, AZM, LZD	*sodA, gelE, ace*	*VanA*
		8	C, P, IPM, VA, RD, TE	*sodA, gelE, ace*	*VanA*
	Pastrami slices (27 isolates)	1	C, P, IPM, VA, RD, SXT, AZM, LZD, AMX	*sodA, gelE, ace*	*VanA*, *VanB*
		4	C, P, IPM, VA, RD, SXT, AZM, LZD, AMX	*sodA, gelE, ace*	*VanA*
		3	C, P, IPM, VA, RD, SXT, TE, LZD, AMX	*sodA, gelE, ace*	*VanA*, *VanB*
		2	C, P, IPM, VA, RD, SXT, TE, LZD, AMX	*sodA, gelE, ace*	*VanA*
		1	C, P, IPM, VA, RD, SXT, TE, LZD, AMX	*sodA, gelE, ace*	*VanB*
		2	C, P, IPM, VA, RD, SXT, TE, LZD	*sodA, gelE, ace*	*VanA*
		2	C, P, IPM, VA, RD, SXT, TE, LZD	*sodA, gelE*	*VanA*
		1	P, IPM, VA, RD, LZD, CN	*sodA, gelE, ace*	*VanA*, *VanB*
		5	C, P, IPM, VA, RD, LZD, CN	*sodA, gelE, ace*	*VanA*
		1	C, P, IPM, VA, RD, SXT	*sodA, gelE, ace*	*VanA*, *VanB*
		5	C, P, IPM, VA, RD, SXT	*sodA, gelE, ace*	*VanA*
***E***. ***faecium*** (n = 171)	Shawarma sandwiches (57 isolates)	5	C, P, RD, VA, AZM, TE, SXT, LEV, NA, CIP, LZD, AMX	*sodA, gelE, ace*	*VanA*, *VanB*
		7	C, P, RD, VA, AZM, TE, SXT, LEV, NA, CIP, LZD, AMX	*sodA, gelE, ace*	*VanA*
		7	C, P, RD, VA, AZM, TE, LEV, NA, CIP, CN, AMX	*sodA, gelE, ace*	*VanA*
		1	C, P, RD, VA, AZM, TE, LEV, NA, CIP, CN, AMX	*sodA, gelE*	*VanA*, *VanB*
		3	C, P, RD, VA, AZM, TE, LEV, NA, CIP, CN, AMX	*sodA, gelE*	*VanA*
		1	C, P, RD, VA, TE, SXT, LEV, NA, CIP, CN, AMX	*sodA, gelE*	*VanA*, *VanB*
		4	C, P, RD, VA, TE, SXT, LEV, NA, CIP, CN, AMX	*sodA, gelE*	*VanA*
		1	C, P, RD, VA, TE, SXT, LEV, NA, CIP, CN	*sodA, gelE, ace*	*VanA*, *VanB*
		5	C, P, RD, VA, TE, SXT, LEV, NA, CIP, CN	*sodA, gelE, ace*	*VanA*
		2	C, P, RD, VA, AZM, SXT, NA, CIP, LZD	*sodA, ace*	*VanA*
		1	C, P, RD, VA, AZM, SXT, NA, CIP, LZD	*sodA*	*VanB*
		9	C, P, RD, VA, AZM, SXT, NA, CIP, LZD	*sodA*	*VanA*
		11	C, P, AZM, NA, LZD	*sodA*	-
	Hawawshi (51 isolates)	10	C, P, RD, VA, AZM, TE, SXT, LEV, NA, CIP, CN, AMX	*sodA*	*VanA*
		1	C, P, RD, VA, AZM, TE, SXT, LEV, AMX	*sodA, gelE, ace*	*VanA*, *VanB*
		8	C, P, RD, VA, AZM, TE, SXT, LEV, AMX	*sodA, gelE, ace*	*VanA*
		2	C, P, RD, VA, AZM, TE, SXT, LEV, AMX	*sodA, gelE*	*VanA*
		6	C, P, RD, VA, AZM, TE, SXT	*sodA, ace*	*VanA*
		4	C, P, RD, VA, AZM, TE, SXT	*sodA*	*VanA*
		2	C, P, RD, VA, TE, SXT, CIP	*sodA, gelE, ace*	*VanA*, *VanB*
		8	C, P, RD, VA, TE, SXT, CIP	*sodA, gelE, ace*	*VanA*
		10	C, P, RD, AZM	*sodA*	-
	Pastrami slices (63 isolates)	2	C, P, RD, VA, AZM, LEV, NA, CIP, CN, AMX	*sodA, ace*	*VanA*, *VanB*
		5	C, P, RD, VA, AZM, LEV, NA, CIP, CN, AMX	*sodA, ace*	*VanA*
		1	C, P, RD, VA, AZM, LEV, NA, CIP, CN, AMX	*sodA*	*VanA*, *VanB*
		5	C, P, RD, VA, AZM, LEV, NA, CIP, CN, AMX	*sodA*	*VanA*
		12	C, P, RD, AZM, TE, LEV, NA, CIP, CN, AMX	*sodA*	-
		4	C, P, RD, VA, AZM, TE, SXT, CN, AMX	*sodA, gelE, ace*	*VanA*, *VanB*
		8	C, P, RD, VA, AZM, TE, SXT, CN, AMX	*sodA, gelE, ace*	*VanA*
		1	C, P, RD, VA, TE, SXT, LEV	*sodA, gelE, ace*	*VanA*
		9	C, P, RD, VA, TE, SXT, LEV	*sodA, gelE*	*VanA*
		3	C, P, RD, VA, TE, SXT, LEV	*sodA, gelE*	*VanB*
		3	C, P, RD, VA, SXT, LZD	*sodA, gelE, ace*	*VanA*, *VanB*
		10	C, P, RD, VA, SXT, LZD	*sodA, gelE, ace*	*VanA*

*C* Chloramphenicol, *P* Penicillin, *IMP* Imipenem, *VA* Vancomycin, *RD* Rifampin, *SXT* Trimethoprim/Sulfamethoxazole, *TE* Tetracycline, *AZM* Azithromycin, *LZD* Linezolid, *CIP* Ciprofloxacin, *CN* Gentamicin, *LEV* Levofloxacin *NA* Nalidixic acid, *AMX* Amoxicillin.

The present result of VRE is markedly higher than those reported by other researchers worldwide. Holman et al. in Canada^59^, Yılmaz et al. in Turkey^40^, and Klibi et al. in Tunisia^26^ could not detect *vanA* or *vanB* genes among *Enterococcus* isolates collected from meat samples. Interestingly, all (100, 93/93) VRE *faecalis* isolates harbored the *gelE* gene, and 94.6% (88/93) of them had the *ace* gene, while 67.4% (93/138) of VRE *faecium* isolates harbored the *gelE* gene and 61.6% (85/138) of them had the *ace* gene. Notably, all (100%, 231/231) VRE isolates were resistant to ampicillin, imipenem, and vancomycin (Table 4). This highlights the major public health risk of VRE isolates and emphasizes the critical necessity for applying suitable control actions to control their spread.

### Effect of extra heat treatment of RTE meat products on *Enterococcus* spp

**Fig. 7:**
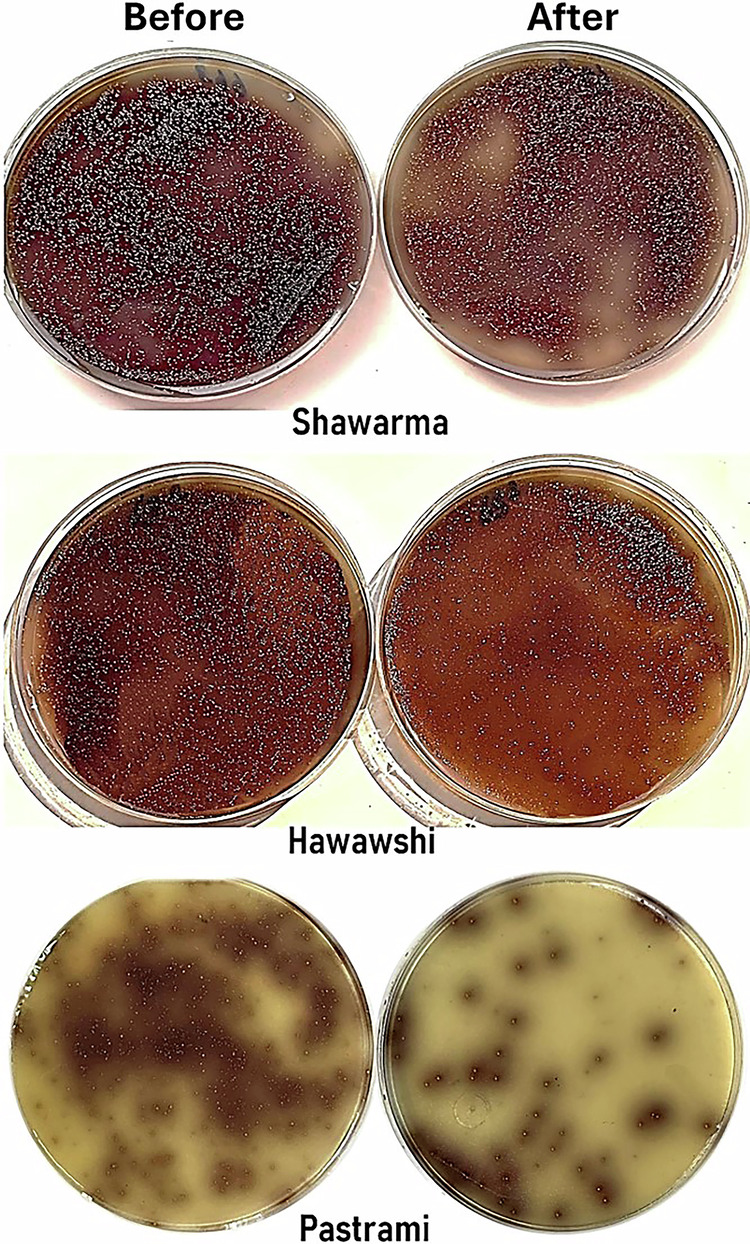
Enterococcus counts on Bile Esculin Azide agar plates from RTE meat product samples before and after microwave heating. A volume of 0.1 mL of the 10^-5^ diluted meat samples was spread onto the plates and incubated for 24 h at 37 °C. No significant difference was observed between RTE meat samples pre- and post-microwave heating.

Although shawarma sandwiches, Hawawshi, and sliced pastrami are ready-to-eat meat products without further heating, an extra heat treatment was applied in the current study to check the effect of microwave heating (220 V, 2450 MHz, 1200 W) for 5 minutes on the survival of *Enterococcus* that passed the regular cooking and preparation of such RTE products in restaurants and markets. Amazingly, the counts of *Enterococcus* colonies cultured on Bile Esculin Azide agar plates were extremely high and exhibited no significant difference in the *Enterococcus* counts in RTE meat products before and after microwave heating (Fig. 7).

The *Enterococcus* counts (log_10_ CFU/g) before and after microwave heating were 8.23 versus 7.86 in shawarma sandwiches, 7.59 versus 7.23 in Hawawshi, and 7.36 versus 6.72 in pastrami, indicating negligible reductions of 0.37, 0.36, and 0.64 logs after 5 min of microwave heating. Despite microwave heating for 5 minutes, *Enterococcus* spp. survived in significant numbers, indicating remarkable resistance to high temperatures. Furthermore, two surviving *Enterococcus* isolates (one *E*. *faecalis* and one *E*. *faecium*) from each product were randomly selected and tested for antibiotic resistance and for the presence of virulence and resistance genes (Table 5). All (6/6) isolates were resistant to the key drugs for enterococcal infections, namely amoxicillin, vancomycin, and linezolid (Table 5). Among the six enterococcal isolates, all (100%, 6/6) harbored the *ace* gene, while 5 (83.3%) harbored the *gelE* gene. Moreover, *vanA* was found in 6 (100%) of the isolates, while 2 (33.3%) of these six *Enterococcus* isolates harbored both *vanA* and *vanB* genes (Table 5). Notably, one *E*. *faecalis* of the 6 heat-resistant enterococcal isolates (1/6; 16.7%) was pan-drug resistant, 33.3% (2/6) were classified as extensively drug-resistant, while 50% (3/6) were categorized as multidrug-resistant (Table 5).

**Table 5:** Antimicrobial resistance profile and molecular characterization of *Enterococcus* isolates (n = 6) recovered from RTE meat products after an additional thermal treatment using a microwave for 5 minutes

RTE meat product	Isolate spp.	Antimicrobial resistance pattern	MAR index	Classification of isolates	Virulence genes	Resistance gene
Shawarma sandwiches	*E*. *faecalis*	C, P, IPM, VA, RD, TE, AZM, CIP, CN, LEV, NA, AMX	0.857	XDR	*sodA*, *gelE*, *ace*	*vanA*
	*E*. *faecium*	C, P, IPM, RD, VA, AZM, TE, SXT, LEV, NA, CIP, LZD, AMX	0.929	XDR	*sodA*, *gelE*, *ace*	*vanA*
Hawawshi	*E*. *faecalis*	C, P, IPM, VA, RD, SXT, TE, AZM, LZD, CIP, CN, LEV, NA, AMX	1.00	PDR	*sodA*, *gelE*, *ace*	*vanA*
	*E*. *faecium*	C, P, IPM, VA, RD, SXT, TE, AZM, CIP, LEV, AMX	0.786	MDR	*sodA*, *gelE*, *ace*	*vanA*,*vanB*
Pastrami slices	*E*. *faecalis*	C, P, IPM, RD, VA, AZM, LEV, NA, CIP, CN, AMX	0.786	MDR	*sodA*, *ace*	*vanA*
	*E*. *faecium*	C, P, IPM, RD, VA, AZM, TE, SXT, CN, AMX	0.714	MDR	*sodA*, *gelE*, *ace*	*vanA*,*vanB*

*C* Chloramphenicol, *P* Penicillin, *IMP* Imipenem, *VA* Vancomycin, *RD* Rifampin, *SXT* Trimethoprim/Sulfamethoxazole, *TE* Tetracycline, *AZM* Azithromycin, *LZD* Linezolid, *CIP* Ciprofloxacin, *CN* Gentamicin, *LEV* Levofloxacin; *NA* Nalidixic acid, *AMX* Amoxicillin, *PDR* Pan-drug resistant, *XDR* Extensively drug-resistant, *MDR* Multidrug-resistant.

These findings underscore the public health concern posed by *E*. *faecalis* and *E*. *faecium*, as the isolates demonstrated tolerance to restaurant processing and microwave heating, in addition to exhibiting resistance to multiple antibiotics and harboring virulence and resistance genes, which collectively exacerbate their pathogenic potential. Their persistence in RTE meat products, where thermal processing is assumed to ensure safety, raises serious concerns regarding consumer exposure. The combination of thermal tolerance and extensive antimicrobial resistance complicates both food safety control measures and clinical management, indicating that infections caused by such resilient enterococci may become increasingly difficult to treat and prevent. Consequently, their presence in ready-to-eat foods highlights the urgent need for improved control strategies and alternative therapeutic approaches.

In conclusion, this study provides compelling evidence that ready-to-eat meat products marketed in Mansoura, Egypt, can serve as a reservoir for highly resilient *E*. *faecalis* and *E*. *faecium*. Beyond their mere presence, the convergence of virulence determinants, extensive antimicrobial resistance, including resistance to last-line agents, such as amoxicillin, vancomycin, and linezolid, and pronounced heat tolerance highlights a critical failure point at the interface between food safety and public health. The ability of these enterococci to survive microwave reheating challenges the common assumption that routine consumer practices are sufficient to mitigate microbial risks in RTE foods. From a public health perspective, these findings underscore the potential for RTE meat products to contribute to the dissemination of difficult-to-treat enterococcal strains within the community. This study, therefore, shifts the concern from contamination alone to the broader issue of pathogen persistence and resilience within food systems. Addressing this risk will require coordinated interventions, including stricter control of antimicrobial use in food animals, enhanced processing and hygiene standards, and the development of alternative control strategies capable of targeting thermotolerant drug-resistant enterococci. Further investigation into the survival mechanisms and transmission pathways of these organisms is essential to inform more effective risk management approaches for RTE foods.

## Methods

### Collection of samples

In the present study, a total of 135 ready-to-eat meat products (45 shawarma sandwiches, 45 Hawawshi sandwiches, and 45 pastrami slices) were purchased from different restaurants and markets around Mansoura city, especially those present in Mansoura University Food Court, in the period between July 2023 and January 2024. Each meat sample was placed in aseptic conditions in a sterile plastic bag in a cooled container. Then, meat product samples were taken to the laboratory of Meat Hygiene and Control, Food Hygiene and Control Department, Faculty of Veterinary Medicine, Mansoura University, where they were examined for the presence of *Enterococcus* spp. using standard microbiological techniques.

### Isolation and identification of *Enterococcus*

For the preparation of meat samples, 10 grams from each sample were homogenized in a laboratory stomacher (Seward Laboratory, London, UK), with 90 mL sterilized buffered peptone water (Oxoid CM0509). For enrichment, 10 mL of the homogenized solution were transferred into a flask containing 100 mL of de Man, Rogosa & Sharpe broth (MRS, Oxoid, Basingstoke, UK) and incubated at 37 °C for 24 h. Serial dilutions were prepared from the overnight enriched broth samples, and 0.1 mL of the appropriate dilution (10^−4^, 10^−5^, and 10^−6^) was spread on Bile Esculin Azide agar (M493I; HiMedia, India) plates and incubated at 37 °C for 24 h. Presumptive *Enterococcus* colonies on Bile Esculin Azide agar plates are small, transparent colonies with brown-black haloes. Any colony that grows on the media without blackening of the media was considered negative^60^. Typical colonies were subjected to further confirmation by both biochemical identification and molecular confirmation. Biochemical tests, including catalase test, oxidase test, detection of hemolysis, growth at 6.5% NaCl, growth at 10 °C and 45 °C, Hippurate hydrolysis test, and bile esculin test, were performed on all presumptive isolates to identify the *Enterococcus* species. Biochemically identified *Enterococcus* isolates were further molecularly verified and differentiated into *E*. *faecalis* and *E*. *faecium*.

### Antimicrobial susceptibility testing of isolated *E*. *faecalis* and *E*. *faecium*

Antimicrobial susceptibility patterns of the 270 isolates were tested against fourteen antibiotics related to eleven different classes using the Kirby-Bauer disk diffusion method on the Mueller–Hinton agar (MH; CM0337; Oxoid Ltd., Basingstoke, UK) according to the Clinical and Laboratory Standards Institute guidelines^61^ except for vancomycin, which was performed with the agar dilution method. Enterococci with a MIC breakpoint of 4 µg/mL are considered susceptible, 8–16 µg/mL is considered intermediate, and ≥ 32 µg/mL are considered vancomycin-resistant enterococci (VRE)^61^. The following antibiotics were examined: Aminoglycosides (Gentamicin, CN—120 μg) for high-level aminoglycoside resistance (HLAR) testing, Quinolones (Nalidixic acid, NA—30 μg; Ciprofloxacin, CIP—5 μg; Levofloxacin, LEV—5 μg), Penicillins (Penicillin G, P—50 μg; Amoxicillin—10 μg), Sulfonamides (Trimethoprim/Sulfamethoxazole, SXT—25 μg), Tetracyclines (Tetracycline, TE—30 μg), Glycopeptides (Vancomycin), Oxazolidinones (Linezolid, LZD—10 μg), Carbapenems (Imipenem, IPM—10 μg), Phenicols (Chloramphenicol, C—30 μg), Ansamycins (Rifampin, RA—30/10 μg), and Macrolide (Azithromycin, AZM—15 μg). Erythromycin interpretive was used as a representative macrolide, as interpretive breakpoints for azithromycin are not available for *Enterococcus* spp. Antimicrobial susceptibility results were interpreted according to CLSI M100 guidelines. Only antibiotics with established interpretive criteria for *Enterococcus* spp. were included in the categorical analysis.

*Enterococcus* isolates were classified as multidrug-resistant (MDR) if they demonstrated resistance to one or more antibiotics across at least four antibiotic classes. They were categorized as extensively drug-resistant (XDR) if they were resistant to all but one or two of the tested antibiotic classes, while those showing resistance to all tested antibiotics in every class were designated as pan-drug-resistant (PDR)^62^. The multiple antibiotic resistance (MAR) index for the identified *Enterococcus* isolates was calculated by dividing the number of antibiotics to which each isolate was resistant by the total number of antibiotics tested^63^. A MAR index value greater than 0.2 indicates the overuse and misuse of antimicrobials, while a MAR index value of > 0.4 is usually from human fecal origin^64^

### Molecular confirmation and differentiation of *Enterococcus* spp

The genomic DNA from each suspected *Enterococcus* isolate was extracted using the Gene JET Genomic DNA Purification Kits (Roche Applied Science, Germany, Cat. No. 11 796 828 001), according to the manufacturer’s instructions. Genomic DNA from the *E. faecalis* ATCC 29212 strain and the *E. faecium* ATCC 19434 strain was prepared as control-positive templates, whereas genomic DNA from the *E. coli K-12 DH5α* strain was used as a negative control template for PCR analyses.

All the 270 biochemically positive *Enterococcus* isolates (90 each from shawarma sandwiches, Hawawshi, and pastrami; 2 isolates from each positive sample) were further verified and differentiated into *E. faecalis* and *E. faecium* by PCR for detection of superoxide dismutase; *sodA* (*E. faecalis*) and *sodA* (*E. faecium*) specific marker genes using their primer sets and cycling conditions^65^ (Table 6).

**Table 6:** Oligonucleotide primer sequences used for the identification of marker, virulence genes, and antimicrobial resistance genes of *Enterococcus faecalis* and *E*

Target gene	Oligonucleotide pimer sequence (5′ → 3′)		PCR amplification cycles	Amplicon size (bp)	References
***SodA*** **(*E. faecalis*)**	Forward: Reverse:	ACTTATGTGACTAACTTAACC TAATGGTGAATCTTGGTTTGG	95°C 2 min; 35 x: 95°C 30 s, 54°C 60 s, 72°C 22 s; 72°C 5 min	360	Jackson et al.^65^
***SodA*** **(*E. faecium*)**	Forward: Reverse:	GAAAAAACAATAGAAGAATTAT TGCTTTTTTGAATTCTTCTTTA	95°C 2 min; 35 x: 95°C 30 s, 50°C 60 s, 72°C 13 s; 72°C 5 min	215	
***gelE***	Forward: Reverse:	ACCCCGTATCATTGGTTT ACGCATTGCTTTTCCATC	95°C 2 min; 35 x: 95°C 30 s, 58°C 60 s, 72°C 25 s; 72°C 5 min	419	Eaton and Gasson^66^
***ace***	Forward: Reverse:	GGAATGACCGAGAACGATGGC GCTTGATGTTGGCCTGCTTCCG	95°C 2 min; 35 x: 95°C 30 s, 56°C 60 s, 72°C 40 s; 72°C 5 min	616	Creti et al.^67^
***vanA***	Forward: Reverse:	ATTGCTATTCAGCTGTACTC GGCTCGAGTTCCTGATGAAT	95°C 2 min; 35 x: 95°C 30 s, 63°C 45 s, 72°C 35 s; 72°C 5 min	559	Seo et al.^68^
***vanB***	Forward: Reverse:	AACGGCGTATGGAAGCTATG CCATCATATTGTCCTGCTGC	95°C 2 min; 35 x: 95°C 30 s, 60°C 45 s, 72°C 30 s; 72°C 5 min	467	

*faecium* isolates recovered from RTE meat products

### Characterization of virulence and antimicrobial resistance genes in *Enterococcus* spp

Additionally, the genes encoding the virulence factors, namely gelatinase (*gelE*) and the collagen-binding proteins (*ace*), were detected in the 270 *Enterococcus* isolates by PCR using specific primer sets of the *gelE* gene^66^ and *ace* gene^67^ at the cycling conditions described in Table 6. The *Enterococcus* isolates exhibiting phenotypic resistance to vancomycin by agar dilution method were further analyzed using PCR to target the vancomycin-resistant genes, *vanA* and *vanB*, using the primer sets and cycling conditions^68^ described in Table 6.

PCR amplification of the identification markers, virulence genes, and vancomycin-resistant genes was carried out using a SimpliAmp™ Thermal Cycler (Thermo Fisher Scientific Inc.). A final PCR reaction volume of 25 μL was prepared, consisting of 12.5 μL of DreamTaq TM Green Master Mix (2X) (Fermentas, Inc., Hanover, MD, USA), 1.0 μL of each primer at 10 pmol (Sigma-Aldrich, Co., St. Louis, MO, USA), 2 μL of the genomic DNA template, and DNase/RNase-free water to reach a total of 25 μL. Positive and negative controls were incorporated in all PCR experiments. An aliquot (8 μL) of each amplified PCR product was subjected to electrophoresis on a 1.5% agarose gel containing 0.5 μg/mL ethidium bromide (Sigma-Aldrich, Co., St. Louis, MO, USA) using 1X TBE running buffer for 50 minutes at a voltage of 95 V. The resulting bands were visualized and captured with an ultraviolet transilluminator. GeneRuler 100 bp DNA Ladder (Thermo Fisher Scientific Inc.) served as a molecular size marker to assess the molecular weights of the PCR products.

### Effect of extra cooking of RTE meat products on the survival of *Enterococcus* spp

To study the effect of extra heat treatment (microwave cooking) on the *Enterococcus* naturally present in RTE meat samples, three independent trials were carried out using microwave cooking of three samples from each tested RTE meat product (Shawarma sandwich, Hawawshi, and pastrami slices) purchased from different restaurants and markets in Mansoura City, Egypt. Each meat sample was divided into two portions (50 grams each) using a sterile knife. The first portion was kept as a control without further microwave heating. The other portion was subjected to microwave treatment (220 V, 2450 MHz, 1200 W) for 5 minutes to investigate the effect of such extra heat treatment on the survival of *Enterococcus* spp. The microbial count of *Enterococcus* spp. was determined before and after microwave cooking. Both microwave-heated and control samples (25 g each) were subjected to homogenization in 225 mL of buffered peptone water, followed by cultivation of 0.1 mL from the appropriate serial dilutions on Bile Esculin Azide agar plates. The plates were incubated at 37 °C for 24 h, and the *Enterococcus* populations were counted and expressed as log_10_ CFU/g.

### Statistical analysis

The Chi-square test was applied to evaluate the differences in antimicrobial resistance rates between *E*. *faecalis* and *E*. *faecium*, as well as to compare the virulence and resistance gene distribution between both *Enterococcus* species. A *P*-value < 0.05 was considered statistically significant. The Student’s t-test was applied to compare the mean counts of the *Enterococcus* population between the microwave-heated RTE meat groups and the non-microwave-heated groups. The difference between the means is statistically significant at *P* < 0.0 and P < 0.01.

## Data Availability

All data supporting the findings of this study are included within the article. No datasets were generated or analyzed during the current study. Any additional information is available from the corresponding author upon reasonable request.

## Code availability

No applicable.
